# Strategies to Improve the Efficiency of Somatic Cell Nuclear Transfer

**DOI:** 10.3390/ijms23041969

**Published:** 2022-02-10

**Authors:** Kanokwan Srirattana, Masahiro Kaneda, Rangsun Parnpai

**Affiliations:** 1 Embryo Technology and Stem Cell Research Center, School of Biotechnology, Institute of Agricultural Technology, Suranaree University of Technology, Nakhon Ratchasima 30000, Thailand; kanokwan.sri@sut.ac.th; 2 Laboratory of Veterinary Anatomy, Division of Animal Life Science, Tokyo University of Agriculture and Technology, Tokyo 183-8509, Japan; kanedam@cc.tuat.ac.jp

**Keywords:** cloning efficiency, embryo, epigenetic modification, nuclear reprogramming, somatic cell nuclear transfer

## Abstract

Mammalian oocytes can reprogram differentiated somatic cells into a totipotent state through somatic cell nuclear transfer (SCNT), which is known as cloning. Although many mammalian species have been successfully cloned, the majority of cloned embryos failed to develop to term, resulting in the overall cloning efficiency being still low. There are many factors contributing to the cloning success. Aberrant epigenetic reprogramming is a major cause for the developmental failure of cloned embryos and abnormalities in the cloned offspring. Numerous research groups attempted multiple strategies to technically improve each step of the SCNT procedure and rescue abnormal epigenetic reprogramming by modulating DNA methylation and histone modifications, overexpression or repression of embryonic-related genes, etc. Here, we review the recent approaches for technical SCNT improvement and ameliorating epigenetic modifications in donor cells, oocytes, and cloned embryos in order to enhance cloning efficiency.

## 1. Introduction

Somatic cell nuclear transfer (SCNT) or cloning involves transferring a donor cell into an enucleated oocyte, enabling the reprogramming of terminally differentiated cells into totipotent cells [1]. Since the first cloned sheep, Dolly, was born in 1996 [1], cloned offspring have been successfully produced in 23 mammalian species (reviewed by [2]). SCNT is a unique tool for multiplying genetically valuable animals, wildlife conservation, genetically modified animal production, and biomedical application (reviewed by [3,4]). However, overall cloning efficiency is still low, varying between 0.1 and 16.0% (reviewed by [5,6]). The low percentage of healthy cloned offspring per transferred embryos is a bottleneck for scientific and commercial applications. A number of abnormalities such as chromosomal aberrations, incompatibility between mitochondrial and nuclear genomes, large offspring syndrome, and placental dysfunction-related disorders have been found in cloned embryos, conceptuses, and offspring [2,7,8,9,10,11], which is believed to be caused by incomplete or unfaithful epigenetic remodeling and reprogramming of donor cell nuclei [12,13,14]. Many researchers have investigated the effects of donor cell type, oocyte maturation stage, SCNT protocol, embryo activation method, culture condition, etc. on the developmental potential, embryo quality, pregnancy rate, and birth rate of cloned embryos in order to improve cloning efficiency (reviewed by [15,16]). Optimizing these cloning parameters could increase cloning efficiency, but it remains low. These strategies might not be enough to make a significant improvement in cloning efficiency (reviewed by [17]). Notably, aberrant epigenetic modifications have been found in cloned embryos, which affect low cloning efficiency, abnormal cloned embryo phenotype, and low viability cloned animals (reviewed by [17,18]). Epigenetic modifications such as DNA methylation, histone acetylation, histone methylation, genomic imprinting, and X-chromosome inactivation are crucial events for the nuclear reprogramming process (reviewed by [15,19]). Theoretically, epigenetic modifications of the donor cell nuclei or cloned embryo could increase accessibility for reprogramming [20].

Epigenetic modifications such as DNA methylation and histone modifications are key factors to regulate gene expression, and they play an important role on embryonic development [21,22]. The developmental efficiency to the blastocyst stage of cloned embryos depends on the donor cell’s ability to reprogram its genome to that of a zygote. Differentiated bovine somatic cells and cloned embryos had higher levels of DNA methylation than gametes and early embryos produced in vivo [23]. Methylation at the 5-position of cytosine (5mC) is an important epigenetic modification during mammalian embryo development as it is involved in regulating gene expression, genomic imprinting, transposon silencing, and X chromosome inactivation (reviewed by [24]). DNA methylation is established and maintained by DNA methyltransferases (DNMTs) and erased by ten-eleven translocation (TET) proteins [25]. Treatment with DNA methyltransferase inhibitors (DNMTi) could reduce the DNA methylation level. Moreover, the histone acetylation level of cloned embryos was significantly lower and different from that of in vitro fertilized (IVF) embryos in cattle, rabbits, and pigs [26,27,28]. Histone acetylation is regulated by histone acetyltransferase (HAT) and histone deacetylase (HDAC) enzymes. HAT opens up chromatins, which increases the accessibility of transcriptional factors and epigenetic modifiers to DNA. HDAC stimulates gene silencing [29]. Histone deacetylase inhibitors (HDACi) prevent the removing of acetyl groups from lysine residues of histone, resulting in chromatin still opening up, leading to gene expression. Notably, non-specific modulator agents such as DNMTi and HDACi target the entire chromatin landscape, which globally modulated DNA methylation and histone acetylation, respectively.

In this review, we discussed the current knowledge of improving the SCNT procedure, in vitro maturation (IVM), in vitro culture (IVC) media, modification of donor cells, oocytes and cloned embryos, and amelioration of epigenetic status during SCNT in order to increase cloning efficiency.

## 2. Technical Improvement of SCNT

The traditional SCNT method using micromanipulator was used to produce the first cloned sheep, Dolly [1]. Briefly, the meiotic spindle complex of metaphase II (MII) oocyte is enucleated using a specialized micropipette under an inverted microscope equipped with a micromanipulator. Then, a single donor cell is transferred into the perivitelline space of the enucleated oocyte by a micropipette. After electrofusion of the transferred donor cell with an enucleated oocyte, reconstructed oocytes are chemically activated and are cultured in the culture media [1]. The traditional SCNT method is well established and has been employed to produce cloned animals by a large number of research groups (reviewed by [17]). SCNT involves a series of procedures including donor cell preparation, oocyte maturation, enucleation, donor cell injection, fusion, activation, embryo culture, and embryo transfer [1]. Suboptimal conditions of any of these steps can have substantial effects on the development of cloned embryos and offspring.

In large animals such as cattle, buffaloes, and pigs, the meiotic spindle complex of MII oocytes is hardly detectable due to the high lipid content in the cytoplasm. MII spindles are normally identified by fluorescence staining (Hoechst 33342) and visualized under ultraviolet (UV) light [1]. However, exposing with UV light has detrimental effects on embryo development. An alternative method is blind enucleation. The 1st polar body (1st PB) and a small volume of cytoplasm underneath the 1st PB are squeezed out. The squeezed-out cytoplasm is stained with Hoechst 33342 and examined under UV light to identify MII spindles. Therefore, an enucleated oocyte is not exposed to UV light [30]. Later, a non-invasive spindle imaging system, polarized light birefringence has been used to enhance the efficiency of enucleation. However, the imaging system is expensive [31]. On the other hand, meiotic spindles of mouse and rat oocytes are easy to identify under phase contrast microscope, and therefore, neither UV light exposure nor expensive equipment is required. Recently, a robotic label-free precise enucleation was established in cloned pigs. This technique reduced the cytoplasmic loss during enucleation by 60% with an enucleation success rate of 95%. The cloned embryos derived from robotic enucleated oocytes had a two times higher cleavage rate than that of blind enucleated oocytes [32].

There are three procedures to transfer and fuse a donor cell with an enucleated oocyte. Firstly, a single donor cell is inserted into the perivitelline space of an enucleated oocyte, after which the donor cell–cytoplast couplets are fused using electric pulses; this method is known as electrofusion [1]. Later, in 1998, the first cloned mouse was produced by a single-step SCNT, which is also known as the Honolulu technique. The donor cell’s nucleus is extracted and is directly injected into an enucleated oocyte using a piezoelectric device [33]. The third technique is a virus-mediated cell fusion using inactivated Sendai virus [34]. This technique was applied as an alternative procedure to induce cell fusion in cloned mice [35].

As described previously, traditional SCNT requires micromanipulation skills and expensive equipment. The handmade cloning (HMC) technique has been established to solve these problems. HMC is a simplified SCNT with no requirement for a micromanipulator [36]. For enucleation, the zona pellucida of the oocyte is removed by enzymatic digestion. Next, the zona-free oocyte is bisected by hand using an ultra-sharp blade under a stereomicroscope. Chromosome-free hemi-cytoplasts are selected by Hoechst 33342 staining. Then, a donor cell is fused with two hemi-cytoplasts to restore the original oocyte volume [37]. HMC has been used to produce cloned animals such as cattle [38,39], horses [40], pigs [41], buffaloes [42], sheep [43], and camels [44]. However, HMC has some disadvantages such as the zona-free embryos might be exposed to toxic substances during IVC, the requirement for a large number of oocytes, as HMC needs two oocytes to make one embryo, and mitochondria heteroplasmy issues. Therefore, a modified handmade cloning (mHMC) technique that does not require the bisection process has been established to solve the oocyte waste during enucleation and mitochondria heteroplasmy. During enucleation, a small volume of cytoplasm containing the chromosome of a zona-free oocyte is removed using a pulled Pasteur pipette under a stereomicroscope [45,46]. Therefore, only one oocyte is required to produce a cloned embryo. mHMC has been used to produce cloned blastocysts in sheep [45], pigs [47], and cloned offspring in goats [48], cattle [49], and camels [50]. The use of different SCNT techniques among different cloning laboratories varies due to animal species, equipment availability, and the technical skills of workers.

Slaughterhouse-derived oocytes are typically used for the large-scale production of cloned embryos in many livestock species. The oocytes are normally selected by the morphological appearance. Staining with Brilliant Cresyl Blue (BCB) was first used as a marker to pre-select porcine oocytes [51]. Growing oocytes have high levels of glucose-6-phosphate dehydrogenase (G6PDH) activity, which can reduce BCB to a colorless state (BCB−). At the end of the oocyte growth phase, G6PDH activity is inactive. Therefore, the fully grown oocytes have blue cytoplasm (BCB+) [52]. BCB staining has been widely used for selecting good quality oocytes in various species, including cattle [53,54], goats [55], horses [56], pigs [57,58], sheep [59,60], buffaloes [61,62], rabbits [63], mice [64], rats [65], dogs [66], cats [67,68], camels [69], and humans [70]. BCB+ oocytes had a higher maturation rate [56,62,63,69,70,71], mitochondrial DNA (mtDNA) copy number [57,58], blastocyst rate [53,54,61,63], calving rate [54], as well as total cell number [54,63] and inner cell mass (ICM) to trophectoderm (TE) ratio [54,61,63] when compared with those of BCB− oocytes. Furthermore, cloned blastocysts derived from BCB+ oocytes had higher levels of histone H3 lysine 18 acetylation (H3K18ac) [54,61], *OCT4*, *SOX2*, and *CDX2* expression [54,61], and also had lower apoptosis cells [54,63]. Therefore, BCB staining can be used as a reliable method for oocyte selection, thus enhancing cloning efficiency.

Numerous research groups have attempted to determine suitable donor cell types and cell synchronization methods in order to improve cloning efficiency. The epigenetic states of donor cells significantly affect the development of cloned embryos. Zhai et al. reported that mesenchymal stem cells (MSCs) from bone marrow had more active epigenetic markers and fewer repressive epigenetic markers than those of fetal fibroblasts. The cleavage and blastocyst rates of cloned porcine embryos derived from MSCs were significantly higher compared with fetal fibroblasts [72]. The effects of donor cells on cloned embryo development have been reviewed by Gouveia et al. [73].

## 3. Impact of Cytoplasmic and Mitochondrial Manipulation

### 3.1. Cytoplasmic and Mitochondrial Supplementation in Enucleated Oocytes

During enucleation, the MII spindle is taken out with a volume of cytoplasm. Cytoplasmic volume plays an important role in the development of cloned embryos. When 50% of the cytoplasm was removed from enucleated bovine oocytes, the cell numbers in the cloned blastocysts were significantly low [74]. An increase in cytoplasmic volume by fusion or by aggregation had positive effects on embryo development, supporting the establishment of pregnancies and the birth of a viable cloned calf [75]. Hence, cytoplasm supplementation can be used to restore the cytoplasm loss. Cytoplasm injection cloning technology (CICT) is a modification of the traditional SCNT where approximately 30% of the cytoplasm of a donor oocyte is injected into the perivitelline space of an enucleated oocyte. CICT can increase cytoplasmic volume of an enucleated oocyte without removing the zona pellucida [76]. Additional cytoplasm increased the blastocyst formation rate, embryo quality, and improved epigenetic reprogramming by decreasing expression levels of DNA methylation-related genes (*DNMT1* and *DNMT3a*) of cloned bovine embryos [76]. Similarly, in cloned mice, cytoplasmic supplementation improved the in vitro development and quality of cloned mouse embryos. Additionally, CICT-derived mouse embryos had a lower number of apoptotic cells and higher acetylation levels when compared with those of SCNT-derived embryos [77]. Notably, CICT significantly increased the birth rate of cloned cat embryos when compared to that of SCNT (4.8% and 0.7%, respectively) [78]. In contrast, cytoplasm supplementation could not improve cloned porcine embryo development in regard to the blastocyst rate, total cell number, and apoptotic cells. The transcription levels of embryonic lineage differentiation genes (*OCT4* and *CDX2*), pro-apoptotic genes (*BAX* and *BAK*), anti-apoptotic gene (*BCL2*), mitochondria activity-related genes (*MFN* and *TFAM*), and methylation-related genes (*DNMT1* and *DNMT3a*) showed no significant differences between cytoplasm supplemented and non-supplemented cloned porcine embryos [79].

Poor-quality and aged oocytes contain low and dysfunctional mitochondria. Mitochondrial supplementation has been shown to positively increase blastocyst rates and the quality of embryos in mice, humans, cattle, and pigs (reviewed by [80]). The combination of mitochondrial supplementation and SCNT (miNT) has been established in a bovine model. Mitochondria was extracted from donor oocytes and then was injected into an enucleated oocyte. Mitochondrial supplementation did not increase the blastocyst formation rate nor the total cell number of cloned bovine embryos. However, miNT-derived blastocysts had a higher mtDNA copy number when compared with that of SCNT. Additional mtDNA led to an increase in the expression of genes involved in the glycolytic metabolism and embryonic development [81]. More studies on mitochondrial supplementation in combination with other assisted reproductive technologies have been recently reviewed by Ferreira et al. [82].

### 3.2. Manipulation of Mitochondrial DNA in the Donor Cells Prior to SCNT

As a whole donor cell is transferred into an enucleated oocyte during SCNT, not only is the nuclear genome passed onto the oocyte but also mitochondria accompanying the donor cell are transferred as well [83,84]. There are three possibilities for the fate of mtDNA in cloned embryos: (1) mtDNA is only inherited from the oocyte, while donor cell mtDNA is either not inherited or is eliminated, (2) mtDNA is inherited from the donor cell while oocyte mtDNA is eliminated, and (3) oocyte and donor cell mtDNA co-exist. The world’s first cloned offspring, Dolly, and the other first set of nine cloned sheep inherited their mtDNA from the oocyte. None of the donor cell mtDNA was found in the examined tissues [85]. Oocyte-only mtDNA was also found in cloned bovine embryos and offspring [86]. On the other hand, the co-existence of oocyte and donor cell mtDNA was reported in cloned bovine offspring. The percentage of donor cell mtDNA in the offspring ranged from 0.4 to 4% [87]. High levels of donor cell mtDNA transmission (40–47%) have been found in cloned sheep, bovine, and porcine offspring [84,88,89]. In cloned cattle, donor cell mtDNA was found in 54 out of the 60 embryos, three out of the ten fetuses, and three out of the six healthy offspring [90]. Notably, mtDNA from both panda donor cell and rabbit oocyte co-existed at the blastocyst stage of panda–rabbit interspecies SCNT embryos. Panda donor cell mtDNA was detected in fetuses, but the rabbit oocyte mtDNA has been eliminated. However, no live offspring were produced [91]. Previous reports showed that the mtDNA present in somatic cells is likely to have accumulated deletions or mutations as part of the aging process [92,93]. To avoid any confounding outcomes and donor cell mtDNA transmission, mtDNA was depleted from the bovine donor cells by mtDNA depleting agent (2′,3′-dideoxycytidine, ddC). The ddC did not have detrimental effects on chromosomal DNA integrity, as the chromosomes of mtDNA-depleted cells did not exhibit any deletions or duplications when compared with non-depleted cells. Cloned bovine embryos derived from mtDNA-depleted cells harbored only oocyte mtDNA. The mtDNA depletion of the donor cells combined with HDACi (trichostatin A, TSA) treatment of cloned embryos positively modulated the expression levels of genes involved in DNA methylation, embryonic formation, and embryonic development. Taken together, depleting mtDNA from the donor cells prior to SCNT not only prevents the transmission of donor cell mtDNA but also positively modulates the gene expression patterns of cloned bovine embryos [94]. Notably, post-implantation development, calving rate, and the health of the offspring from cloned embryos derived from mtDNA-depleted donor cells need to be determined.

## 4. Ameliorate Global DNA Methylation and Histone Acetylation Using DNA Methyltransferase and Histone Deacetylase Inhibitors

Several strategies have been used to aid epigenetic reprogramming to get normal DNA methylation and chromatin remodeling, which would improve the cloning efficacy as described above. Notably, the first technical breakthrough of HDACi treatment on SCNT was performed by Kishigami et al. in 2006. The cloned mouse embryos were treated with 5–50 nM TSA for 10 h from the time point of oocyte activation after SCNT [95]. TSA not only increased in vitro embryo development but also enhanced the live birth rate in cloned mice [95,96,97]. Furthermore, adult ICR mice (an outbred strain), which have never been directly cloned, were successfully produced for the first time when TSA was used [97]. Notably, TSA treatment increased histone acetylation and reduced repressive histone methylation in cloned mouse embryos [98]. These remarkable outcomes gave rise to many researchers to apply TSA with other species. TSA became the most commonly used to improve cloning efficiency in rhesus monkeys [99], pigs [100], rabbits [27], cattle [101], buffaloes [102], and cynomolgus monkeys [103] with optimal concentration and exposure time. The toxicity of TSA can be found at high concentration and long exposure time [104,105]. In recent years, a vast majority of the studies focused on the use of TSA-dependent epigenomic modulation for SCNT in mammals have been carried out to epigenetically modify porcine donor somatic cells [106], donor stem cells [107], activated SCNT-derived oocytes, and resultant embryos [108,109]. Nevertheless, a number of reports showed that TSA had no beneficial effect in cloned cattle [26,110,111,112], sheep [113], and gaur-bovine interspecies SCNT [114]. TSA had a detrimental impact on cloned rabbit embryos [115]. Controversial results may be due to the species-specific difference, time, and concentration of TSA treatment. Although TSA could broadly correct aberrant acetylation in many regions of cloned embryos, some regions remained hypoacetylated [116,117].

Another HDACi that was less toxic, Scriptaid (6-(1,3-dioxo-1H, 3H-benzo[de]isoquinolin-2-yl)-hexanoic acid hydroxyamide), improved cloning efficiency in inbred mice [104], inbred miniature pigs [118], and sheep [119]. Furthermore, Scriptaid was more effective at enhancing the in vitro development in cloned mice and sheep than TSA [104,119]. Scriptaid supplementation in IVC media promoted a two-fold increase in the blastocyst rate of cloned canine embryos [120]. Scriptaid also significantly increased the total cell number of cloned porcine embryos at the blastocyst stage [121]. The supplementation of Bufexamac, an HDACi in porcine IVM media, had no significant effect on the maturation rate, but it increased the histone H3 lysine 9 (H3K9), histone H3 lysine 14 (H3K14), and histone H4 lysine 8 (H4K8) acetylation levels of porcine oocytes. Moreover, Bufexamac treatment significantly increased the blastocyst rate and upregulated the *OCT4* and *CDX2* expression of cloned porcine embryos [122]. Other HDACi such as sodium butyrate (NaBu) [123], m-carboxycinnamic acid bishydroxamide [124], oxamflatin [125], suberoylanilide hydroxamic acid (SAHA) [125], valproic acid (VPA) [126], psammaplin A [127], abexinostat [128], belinostat [129], dacinostat [130], mocetinostat [131], and quisinostat [132] have been used to enhance the reprogramming ability of the donor cells in cloned embryos and, as a consequence, to improve their developmental competences and molecular quality.

When bovine donor cells were treated with a DNMTi, 5-aza-2′-deoxycytidine (5-aza-dC), the global methylation of chromatin in the donor cells was decreased. However, no improvement on the blastocyst rate of cloned bovine embryos was found [23,133]. The treatment of donor cells with *S*-adenosylhomocysteine (SAH), another DNMTi, induced global DNA demethylation and increased the blastocyst rate of cloned bovine embryos as well as increased the telomerase activity in both donor cells and cloned embryos [134]. Moreover, the treatment of donor cells with TSA increased the global histone acetylation levels and increased the blastocyst rate of cloned bovine embryos [133,135,136,137]. NaBu also improved the in vitro development in cloned cattle [135] and rabbits [138]. In contrast, Das et al. found that treatment of cloned porcine embryos with NaBu increased the blastocyst rate; however, no beneficial effect was found when donor cells were treated with NaBu [123]. Fang et al. showed that co-treatment of DNMTi (Zebularine) and HDACi (Scriptaid) in ovine donor cells and cloned embryos increased the blastocyst rate and ameliorated the abnormal expression of embryonic development-related genes (*OCT4*, *SOX2*, *H19*, *IGF2*, and *DNMT1*) [139]. Although the treatment of donor cells with DNMTi and/or HDACi could increase the blastocyst rates in several species, no or a minor beneficial effect on the birth have been reported. These results suggested that DNMTi and/or HDACi treatment should be performed with embryos, not on the donor cells.

The HDACi- and/or DNMTi-based strategies of epigenomic modulation have been comprehensively reviewed by Samiec and Skrzyszowska [140]. Recently, mouse cloning efficiency could be improved by chlamydocin analogues, which are a family of newly designed agents that specifically inhibit Class I and IIa HDACs. The results showed that one of the chlamydocin analogues, Ky-9, strongly promoted the development of cloned mouse embryos to a level similar to that of TSA [141].

## 5. The Use of Non-Chemical and Biological Agents as Epigenetic Modifiers

While chemical agents have been used to improve cloning efficiency, there are some concerns about the safety of these drugs on the health of offspring. Biological agents or non-chemical treatment are alternatives to overcome this issue. Previous reports showed that cytoplasmic extracts from GV stage oocytes improved the cloning efficiency in sheep [142] and also modified histone methylation, histone acetylation, and embryonic development-associated genes (*OCT4* and *NANOG*) in cloned pigs [143].

Vitamin C (L-ascorbic acid), a well-known antioxidant, also acts as an epigenetic modifier. Vitamin C enhanced the activity of the TET protein and promoted the oxidation of 5mC to 5-hydroxymethylcytosine (5hmC) [144]. The treatment of donor cells with vitamin C improved the development of cloned bovine embryos [145]. Vitamin C supplementation to IVC media increased the expression levels of transcription factors (*OCT4*, *SOX2*, and *KLF4*) and the acetylation level of histone H4 lysine 5 (H4K5) as well as increased the full-term development of cloned porcine embryos [146]. Chawalit et al. found that vitamin C increased the blastocyst rate but did not change H3K9 and H3K14 acetylation levels in HMC porcine embryos [147]. In cloned mice, vitamin C significantly increased blastocyst and birth rates. However, no significant effects on the expression of *Oct4* and *Nanog*, H3K14 acetylation, and H3K9 methylation (H3K9me) was found [148]. In cloned sheep, the pre-implantation development of embryos and the 5hmC levels in blastocysts were significantly increased when cloned embryos were directly treated with vitamin C. On the other hand, no positive effects were found when the donor cells were treated with vitamin C [149]. This beneficial role of vitamin C on cloned embryos may be attributed to its antioxidant activity and epigenetic modifying function. A combinational treatment of TSA from 0 to 8 h after activation and vitamin C from 8 to 15 h after activation improved the efficiency of mouse cloning from 0 to 15% and decreased the abnormally high levels of H3K9 trimethylation (H3K9me3) and global DNA methylation in cloned mouse embryos [150]. Notably, the birth rate of cloned mouse embryos treated with a combination of vitamin C and psammaplin A was similar to that of vitamin C or psammaplin A alone. Both vitamin C and psammaplin A improved mice cloning efficiency possibly through different mechanisms, as they do not show an additive effect when combined [148].

The supplementation of bovine IVM media with melatonin (a free radical scavenger) decreased the apoptosis level, recovered the integrity of mitochondria, ameliorated the spindle assembly and chromosome alignment, increased the global H3K9 acetylation levels, reduced the H3K9me levels in bovine oocytes, and enhanced the subsequent development of cloned bovine embryos [151]. In cloned cattle, sperm-borne small RNAs regulate α-tubulin acetylation and epigenetic modification in cloned embryos. Sperm-borne small RNA injection of cloned bovine embryos enhanced the developmental competence and significantly increased the live birth rate and decreased the birth weights of offspring [152]. Similarly, the injection of sperm small RNA into cloned rabbit embryos increased the total cell number and H3K9me3 level as well as decreased the apoptosis index of cloned rabbit blastocysts [153].

## 6. Impact of Histone Methylation

Histone methylation is regulated by histone methyltransferases and histone demethylases, which play important roles at all development stages [154]. H3K9me3, a histone marker of transcriptional repression, is considered a key barrier of cloned embryo development. Using RNA sequencing, the arrested cloned mouse embryos at the two-cell stage cause abnormal gene expression due to the maintenance of H3K9me levels [155]. Aberrant epigenetic reprogramming of H3K9me3 was found in cloned cattle [156,157], mice [158], and rabbits [159], which is a major cause of the developmental failure of cloned embryos [116,160]. Matoba et al. found that there are the regions enriched for H3K9me3 in cloned mouse embryos at the two-cell stage, unlike in IVF embryos, and they named these regions Reprogramming Resistant Regions (RRRs). Notably, the demethylation of H3K9me3 using H3K9me3-specific demethylase *Kdm4d* could reactivate the majority of RRRs and improve cloned mouse efficiency [116]. In cloned sheep, when donor cells were treated with recombinant human KDM4D protein, the levels of H3K9me3 and H3K9 dimethylation (H3K9me2) were both significantly decreased. KDM4D treatment improved the blastocyst rate, blastocyst quality, and expression of developmental genes including *SOX2*, *NANOG*, and *CDX2* in cloned sheep [161]. The reduction of H3K9me in donor cells has been reported to enhance the developmental potential in cloned mice [155,162], sheep [161], and pigs [163].

The injection of H3K9me3-specific demethylase *Kdm4a* mRNA reduced H3K9me3 activity and increased blastocyst rates, resulting in improving the efficiency of nuclear transfer-derived embryonic stem cell (ntESC) production in humans [160] and live offspring in monkeys [103]. Similarly, the injection of *KDM4A* mRNA into cloned porcine embryos effectively decreased the H3K9me3 level and increased the blastocyst rate; however, the addition of *KDM4A* significantly elevated the expression levels of *XIST* (X inactivate specific transcript) in both pre- and post-implantation stage embryos, which may cause post-implantation death in cloned pigs [164].

The overexpression of murine *Kdm4b* (lysine demethylase 4B) in the bovine donor cells reduced H3K9me3 and histone H3 lysine 36 trimethylation (H3K36me3) levels, and it improved the blastocyst formation rate, but it did not increase post-implantation development in cloned bovine embryos [165]. Furthermore, *KDM4B* overexpression in cloned bovine embryos reduced the transcriptional level of H3K4me3 and increased 5mC levels [166]. Liu et al. found that *Kdm4b* and a histone H3 lysis 4 trimethylation (H3K4me3)-specific demethylase (*Kdm5b*) were identified as the key factors for two-cell and four-cell arrest of cloned mouse embryos, respectively. The co-injection of *Kdm4b* and *Kdm5b* mRNAs during SCNT could restore transcriptional profiles by reducing the abnormal methylation levels in cloned embryos and increased blastocyst development as well as the pregnancy and birth rates of cloned mice [155]. Two H3K9-specific demethylase genes, *KDM4D* and *KDM4E*, are related to the active demethylation of H3K9me3 and H3K9me2. *KDM4D* and *KDM4E* were deficiently expressed in cloned bovine embryos at the eight-cell stage. The overexpression of *KDM4E* can restore the global transcriptome, improve blastocyst formation, and increase the efficiency of bovine cloning. Hence, *KDM4E* is also essential for H3K9 demethylation during embryonic genome activation [162].

Previous reports showed that specific histone methyltransferases inhibitors can be used to modulate H3K9 methylation. BIX-01294, a specific inhibitor of G9A (histone-lysine methyltransferase of H3K9), significantly decreased the levels of H3K9me2 and H3K9me in cloned porcine embryos at two-cell and four-cell stages, respectively. BIX-01294 also increased transcriptional expression of the pluripotency genes (*SOX2*, *NANOG*, and *OCT4*) in cloned porcine embryos at the blastocyst stage. Therefore, BIX-01294 enhanced the developmental competence of cloned porcine embryos through improvements in epigenetic reprogramming and gene expression [167]. On the other hand, BIX-01294 treatment of cloned mouse embryos has beneficial effects in terms of correcting abnormal epigenetic modifications but not on in vitro development [168]. The expression levels of *SUV39H1*, *SUV39H2*, *DNMT1*, *DNMT3A*, and *DNMT3B* were abnormally high in cloned porcine embryos when compared with IVF embryos. The treatment of cloned porcine embryos with chaetocin, an H3K9me3-specific methyltransferase inhibitor, significantly increased the embryo developmental rate and expression of pluripotency-related genes [169]. Chaetocin also enhanced epigenetic reprogramming by reducing the H3K9me3 and 5mC levels and restoring the abnormal expression of H3K9me3-specific methyltransferases and DNA methyltransferases [170].

Histone H3 lysine 27 trimethylation (H3K27me3) is another repressive epigenetic mark, which is regulated by enhancer of zeste homolog 2 (*EZH2*) lysine methyltransferases and lysine demethylase 6A and 6B (*KDM6A/6B*). The overexpression of *Kdm6a* improves the efficiency of mouse cloning, while *Kdm6b* did not. However, the knockdown of *Kdm6b* impeded ectopic *Xist* expression and increased mouse cloning efficiency [171]. The treatment of donor cells and cloned embryos with GSK126 (*EZH2* inhibitor) reduced the H3K27me3 levels and enhanced both porcine cloning and iPSCs efficiency. On the other hand, GSK-J4 (*KDM6A/6B* inhibitor) increased the H3K27me3 level but decreased the development of cloned porcine embryos [172]. Deletions of H3K27me3-imprinted genes (*Sfmbt2*, *Jade1*, *Gab1*, and *Smoc1*) in donor cells normalized gene expression patterns and also increased cloned mouse efficiency to 14%. Notably, *Sfmbt2* deletion was the most effective for improving mouse cloning efficiency [173]. Small interfering RNA (siRNA) has been used to knock down H3K9 methyltransferases, *Suv39h1* and *Suv39h2*, in murine donor cells. The knockdown of *Suv39h1/h2* in the donor cells increased the blastocyst formation rate of cloned mouse embryos [116]. Similarly, the knockdown of *SUV39H1/H2* in bovine donor cells decreased H3K9me3, increased H3K9 acetylation (H3K9ac), and repressed DNA methyltransferase gene expression. The knockdown of *SUV39H1/H2* also improved cloned bovine embryo development, and these cloned embryos had a similar pattern of gene expression to the IVF embryos [174]. Although the modification of donor cells prior to SCNT could increase the blastocyst rates in several species, only a few beneficial effects on birth have been reported.

## 7. Impact of DNA Methylation and Chromatin Structure

Ectopic *DNMT1* expression has been believed to cause aberrant methylation in cloned bovine embryos [175]. Previous reports showed that donor cell *DNMT1* expression can inhibit by DNMTi (5-aza-dC [176,177] and RG108 [178]) and gene knockdown [179]. The knockdown of DNA methyltransferases (*Dnmt3a* and *Dnmt3b*) combined with the overexpression of histone demethylases (*Kdm4b* and *Kdm5b*) reduced the global hyper-methylation status and improved the full-term development of cloned mouse embryos [180].

MicroRNAs are short non-coding regulatory RNA molecules that inhibit translation or contribute to mRNA degradation via binding to the 3′UTR of target mRNAs [181]. MicroRNA 148a (miR-148a) overexpression in porcine donor cells prior to SCNT significantly decreased the levels of *DNMT1* expression and global DNA methylation of the donor cells, and it also significantly increased the blastocyst rate, total cell number, and expression levels of *OCT4* and *NANOG* in cloned porcine embryos [182].

Methyl-CpG-binding domain proteins (MBPs) associate with DNA methylation and histone modification, which are the critical changes of somatic cell reprogramming. Methyl-CpG-binding protein 2 (*Mecp2*) expression was significantly low in cloned mouse embryos [183]. The overexpression of *Mecp2* in mouse donor cells increased the blastocyst rate, expression levels of *Oct4* and *Nanog*, and also the 5hmC level, while it decreased the 5mC level. *Mecp2* may promote the activity of ten-eleven translocation 3 (*Tet3*), which mediates active DNA demethylation during mouse pre-implantation embryonic development [183]. Moreover, the overexpression of *TET3* in the donor cells increased blastocyst formation rates and embryo quality in cloned goats [184] and cattle [163]. Additionally, methyl-CpG-binding domain protein 3 (MBD3) is a core component of the nucleosome remodeling and deacetylase (NuRD) complex, which is crucial for pluripotent stem cell differentiation and embryonic development. The overexpression of *MBD3* in cloned porcine embryos increased the blastocyst rate, total cell number, mRNA expression levels, and also decreased the DNA methylation levels of pluripotency genes (*OCT4* and *NANOG*) [185].

In eukaryotes, chromatin is packaged in a hierarchical structure which is associated with many biological processes. Proper higher-order chromatin folding is crucial for gene regulation and chromosome division during mitosis or meiosis (reviewed by [186,187]). The three-dimensional (3D) chromatin structure consists of chromosome territories, chromatin compartments (A/B), Topologically-Associated Domains (TADs), and loops [188]. Proper 3D chromatin structure establishment is an important step during cell fate transition. The aberrant TADs and compartment A/B organization can be found in cloned mouse embryos that was partially caused by H3K9me3 in the donor cells. The injection of *Kdm4d* mRNA could partially rescue un-disassembled H3K9me3-marked TADs in cloned mouse embryos at the two-cell stage [189]. Cohesion is an essential protein complex for loop and TAD formation [190,191,192]. Zhang et al. found that removing cohesion from donor cells prior to SCNT rescued the activation of zygotic genome activation (ZGA) and increased blastocyst rate in cloned mice [193].

The abnormal methylation of imprinted genes is commonly observed in cloned embryos, and it is one of the primary reasons for their abnormal development and high mortality. Primordial germ cell 7 (*PGC7*) maintains the methylation level of imprinted genes by reducing the levels of 5hmC and increasing levels of 5mC during embryonic development. *PGC7* overexpression in donor cells corrected the aberrant methylation patterns of the imprinted genes (*IGF2R*, insulin-like growth factor 2 receptor and *XIST*), reduced the developmental abnormalities in cloned goat embryos, and significantly enhanced both pregnancy and birth rates [194]. Loss of H3K27me3 imprinting causes placental enlargement and a low birth rate of cloned mouse embryos. Correcting the expression of clustered microRNAs within the *Sfmbt2* gene ameliorated the placental phenotype; moreover, the birth rates were increased about two-fold [195]. Complete loss of H3K27me3 imprinting was found in cloned mouse embryos, which caused the postnatal developmental defects [196]. No significant differential expression of H3K27me3-imprinted genes was found in cloned porcine or cloned bovine embryos. This indicated that the H3K27me3-imprinting system may not be conserved in large-animal species [197]. In fact, the loss of imprinting in *Sfmbt2* of cloned mouse embryos contributed to placental overgrowth. However, *SFMBT2* is not an imprinted gene in pig, cattle, and human [198].

## 8. Impact of X Inactivates Specific Transcript Modification

X chromosome inactivation (XCI) is a process in which one of the two X chromosomes in female cells are inactivated during early embryonic development. A long non-coding RNA gene, *XIST*, is responsible for XCI [199]. The ectopic expression of *XIST*, which is one of the major epigenetic errors, has been found in both male and female cloned mice [200], cattle [200,201], and pigs [164]. The abnormal expression of *XIST* may be associated with high neonatal mortality in cloned animals [202]. *XIST* knockout in male porcine donor cells modulated aberrant *XIST* expression and reduced global H3K9me3 in cloned porcine embryos [164]. The knockdown of *Xist* combined with TSA treatment in cloned mouse embryos could improve the birth rate and cloning efficiency [203]; however, the knockdown of *Xist* only works in males [204]. Notably, the knockout of *Xist* on active X chromosome in cloned mouse embryos showed normal global gene expression and resulted in an eight- to nine-fold increase in cloning efficiency in both male and female [200]. Nevertheless, the injection of anti-*XIST* siRNA into male cloned porcine embryos slightly increased embryonic development [205]. Later, Yang et al. reported that the injection of anti-*XIST* short hairpin RNA (shRNA) into cloned porcine embryos at the two-cell stage reduced *XIST* expression and enhanced the developmental ability of cloned embryos derived from male donor cells [206]. Notably, a combination of *Xist* knockout in donor cells and overexpression of *Kdm4d* could increase by more than 20% efficiency of mouse cloning [196].

## 9. Alternative Methods for Cloning Efficiency Improvement

Many studies using other chemical and non-chemical strategies to improve cloning efficiency have been reported as described below.

Granulocyte colony-stimulating factor (G-CSF), a pleiotropic cytokine, belongs to the hematopoietic growth factor family. The level of G-CSF in the follicular fluid is a predictive biomarker of oocyte and embryo developmental competence after IVF and intracytoplasmic sperm injection (ICSI) in humans [207]. The supplementation of human recombinant G-CSF in porcine IVC media significantly enhanced blastocyst rate and the total cell number, and it significantly decreased apoptotic cells in cloned porcine blastocysts. Moreover, the transcriptional levels of anti-apoptosis (*BCL2*)-, proliferation (*PCNA*)-, and pluripotency (*POU5F1*)-related genes were dramatically upregulated [208]. In bovine cloning, follistatin supplementation during the first 72 h of IVC significantly increased both blastocyst rate and *CDX2* expression in cloned blastocysts [209]. In addition, adiponectin (a protein hormone and adipokine) supplementation in IVC media significantly increased cleavage and blastocyst rates as well as the total cell number of cloned porcine embryos. Adiponectin reduced the level of *XBP1* expression and ER stress-related genes, enhanced the expression levels of *NANOG* and *SOX2*, and decreased that of Caspase-3 [210].

Resveratrol (a natural plant-derived antitoxin) treatment of porcine oocytes during IVM increased the maturation rate, blastocyst rate, and the blastocyst cell number in cloned porcine embryos. Resveratrol improved the quality of porcine oocytes by protecting them from oxidative damage and apoptosis [211]. *Rhodiola sachalinensis* is an herb commonly used in traditional Chinese medicine. The supplementation of *R. sachalinensis* aqueous extract (RSAE) to porcine IVM media did not improve the maturation rate, but it significantly increased the intracellular glutathione level in porcine oocytes. Moreover, RSAE enhanced the cleavage and blastocyst rates of cloned porcine embryos [212]. Asiatic acid is a pentacyclic triterpene enriched in the medicinal herb *Centella asiatica*, and it has been suggested to possess free radical scavenging and anti-apoptotic properties. Asiatic acid supplementation during the IVC improved developmental competence and embryo quality in cloned porcine embryos. Asiatic acid not only enhanced intracellular GSH levels but also attenuated mitochondrial dysfunction. Asiatic acid upregulated expression of the antioxidant (*SOD1*)- and the blastocyst formation (*COX2*)-related genes while downregulating expression of the apoptosis (*CASPASE9*)-related gene in cloned porcine blastocysts [213]. Plant-derived nanoparticles are biologically safe and applicable for improving the quality of oocytes and subsequent embryo development. The supplementation of porcine IVM media with modified *Spirulina maxima* pectin nanoparticles (MSmPNPs) improved the oocyte maturation rate, resulted in a higher cleavage rate, blastocyst development, total cell number, and ratio of ICM:TE compared to the untreated group. MSmPNP treatment increased the level of intracellular glutathione (GSH) while reducing the reactive oxygen species (ROS) level, as well as increasing the expression of the pluripotency-associated genes (*POU5F1*, *DPPA2*, and *NDP52*) in cloned porcine blastocysts [214]. As one of the most powerful natural antioxidants, astaxanthin (Ax) has begun to be applied to the field of reproductive biology. Ax treatment was reported to increase the maturation rate of porcine [215] and bovine oocytes [216]. In contrast, the supplementation of Ax in porcine IVM media did not improve the oocyte maturation rate but significantly increased the cleavage and blastocyst rates of cloned porcine embryos. Moreover, Ax enhanced *GDF9* and *POU5F1* expression in cloned porcine embryos [217].

Notably, Reversine (2-(4-morpholinoanilino)-6-cyclohexylamino-purine analogue) is a dedifferentiating agent and was shown to induce cell plasticity and promote the reprogramming of several differentiated cells to multipotent progenitor cells [218,219,220,221]. Additionally, Reversine modulated the acetylation of histone by changing MEK-dependent signaling, which could alter the reprogramming events in cloned embryos [219]. Reversine treatment increased the blastocyst rate of cloned miniature pig embryos, and normal fetuses were obtained after transferring Reversine-treated embryos into recipients. However, no offspring were born [222].

Sodium chloride is one of the main components regulating the osmolality of a culture medium. The changes in the osmolality affect the maturation of oocytes and embryonic development [223,224]. Recently, Lee et al. studied the effects of NaCl concentration in porcine IVM media. The osmotic pressures of IVM media containing 61.6 (low concentration) and 108 mM (normal concentration) NaCl were approximately 220 and 285 mOsm, respectively. A low concentration of NaCl did not improve the maturation rate of porcine oocytes but significantly increased the blastocyst rate of cloned porcine embryos when compared with that of oocytes cultured in normal NaCl concentration [225]. Recently, pulsed electromagnetic fields (PEMF) treatment of cloned buffalo embryos at the beginning of IVC increased the blastocyst rate, decreased the level of apoptosis, and altered the expression levels of pluripotency-, apoptosis-, metabolism-, and stress-related genes [226].

In bovine cloning, the removal of folate (folic acid) from the donor cell culture media decreased the DNA methylation level of the donor cells as well as increased the blastocyst rate of cloned bovine embryos [227]. Alanine (an amino acid that is used in the biosynthesis of proteins) supplementation in porcine IVM media significantly increased cleavage and blastocyst rates after SCNT. Cloned porcine embryos derived from alanine-treated oocytes significantly increased the mRNA expression of *POU5F1* and *FGFR2*, which are associated with oocyte quality and embryonic development [228]. A previous report showed that treatment of aged porcine oocytes with caffeine, a phosphatase inhibitor, can increase the maturation promoting factor (MPF) activity resulting in reducing of spontaneous activation and fragmentation [229]. In cloned sheep, caffeine treatment of oocytes during IVM increased the total cell number of cloned blastocysts [230]. The supplementation of caffeine during oocyte enucleation or post-fusion enhanced the blastocyst rate of cloned porcine embryos by the upregulated expression of *POU5F1*, *SOX2*, and *NANOG* [231]. Manganese supplementation in porcine IVM media significantly increased the blastocyst rate of cloned porcine embryos. However, there was no substantial difference in the cleavage rate and total cell numbers in blastocysts compared to the untreated group. Manganese improved the developmental competence of cloned porcine embryos by increasing GSH and decreasing ROS levels [232].

A canonical WNT (wingless-related mouse mammary tumor viruses) signaling pathway has been reported to inhibit embryonic development [233]. Dickkopf-1 (DKK1) is a secretory inhibitor of the canonical WNT signaling pathway. DKK1 supplementation in the IVC media on day 5 of embryo culture could increase blastocyst formation, conception, and birth rates of HMC river buffalo embryos [234]. Genetic instability, including DNA double-strand breaks (DSBs) and chromosome segregation errors, has been found in both cloned human and cloned mouse embryos, resulting in delayed DNA replication and abnormal mitosis [235]. Rad51 homologous 1 (*RAD51*) is a DNA-binding protein that maintains genome stability and regulates signaling proteins to control the DNA damage response, replication, repair, and recombination (reviewed by [236]). The activity of RAD51 and DSBs were lower in cloned mouse embryos than IVF embryos, which caused a decrease in DNA repair and an increase in genetic instability, resulting in the developmental arrest of cloned embryos [237]. To repair genetic instability or DSBs, cloned mouse embryos were treated with RAD 51-stimulatory compound 1 (RS-1), which is an activator of *Rad51*. RS-1 treatment recovered RAD51 activity, overcame developmental arrest at the two-cell stage, and also increased blastocyst formation and offspring rates in cloned mouse embryos [237]. A combination of *Kdm4a* mRNA injection and RS-1 treatment significantly increased the blastocyst rates of cloned mouse embryos (82.5%) when compared to those of only *Kdm4a* injection (66.0%), only RS-1 treatment (65.9%), and control cloned embryos (35.1%) [237].

ZGA is an important process for donor cell reprogramming in cloned embryos. The transcription factor of double homeobox (*Dux*) was identified as a key inducer of ZGA in normal fertilized embryos [238,239,240]. The transient overexpression of *Dux* improved the cloning efficiency in mice and facilitated fully chemically-induced somatic reprogramming. These cloned embryos also had transcriptome profiling similar to that of IVF embryos. Furthermore, the combination of *Dux* overexpression and *Dnmt3a/3b* knockdown increased the birth rate of cloned mice [241]. The overexpression of *Dux* significantly improved cloned mouse embryo development by correcting the aberrant H3K9ac to overcome two-cell arrest [117]. In addition, the overexpression of full-length *Dux* mRNA in cloned mouse embryos improved the efficiency of pre-implantation development and increased the expression of ZGA- related genes (*Zscan4* and *Mervl*) [242].

## 10. Concluding Remarks

**Figure 1:**
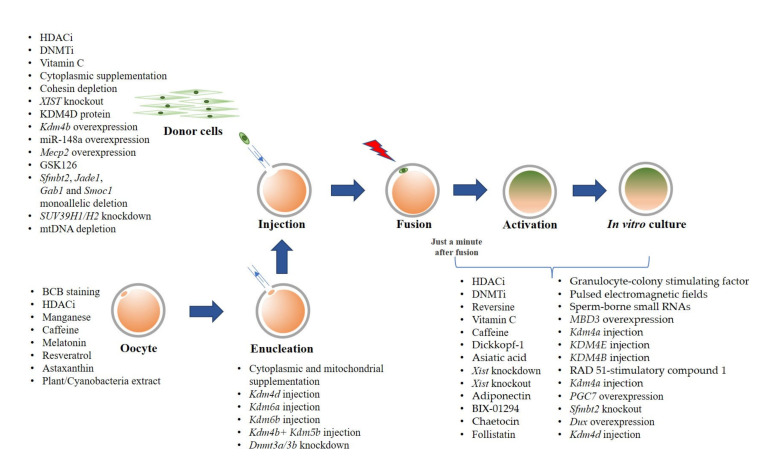
Summary of strategies for cloning efficiency improvement.

We reviewed methods to improve SCNT efficiency by technical optimizations on the SCNT procedure to overcome aberrant epigenetic modifications using chemical and non-chemical treatments, and also targeted modification. Many approaches to assist the nuclear reprogramming have been employed either in donor cells, oocytes, and/or cloned embryos, as summarized in Figure 1. However, significant successes in live birth rates have been mainly reported in mice, while only a few were reported in pigs, cattle, cynomolgus monkeys, and goats (Table 1). Future studies in large animal models are needed. A better understanding on the epigenetic reprogramming is essential to improve overall cloning efficiency. It would also help to develop new approaches to produce cloned animals.

## Figures and Tables

**Table 1:** Summary of cloned offspring produced by using epigenetic modulations.

Species	Modifier	Treatment on	No. of Reconstructed Embryo	No. of 2-Cell (%)	No. of Blastocyst (%)	No. of Embryo Transferred	No. of Pregnant/ Recipient (%)	No. of Offspring	Birth Rate per Embryo Transferred (%)	Reference
mouse	TSA 50 nM/10 h	embryos	178	170 (98.0)	n/a	170	n/a	11	6.5	[95]
	Control		317	305 (96.0)	n/a	297	n/a	1	0.3	
mouse	TSA 100 nM/8 h	embryos	98	n/a	66 (67.0)	356	n/a	10	2.8	[96]
	Control		96	n/a	37 (39.0)	366	n/a	3	0.8	
mouse	TSA 5 nM/10 h	embryos	182	120 (94.0)	n/a	120	n/a	5	4.2	[97]
	Control		155	115 (93.0)	n/a	115	n/a	0	0	
mouse	TSA 100 nM/10 h	embryos	140	129 (92.0)	112 (80.0)	n/a	n/a	6	4.7	[104]
	Control		270	214 (79.0)	74 (35.0)	n/a	n/a	1	0.5	
mouse	Scriptaid 250 nM/10 h	embryos	160	144 (90.0)	116 (81.0)	n/a	n/a	11	7.6	[104]
	Control		270	214 (79.0)	74 (35.0)	n/a	n/a	1	0.5	
mouse	TSA 50 nM/6 h	embryos	89	83 (98.0)	n/a	83	n/a	13	16.0	[125]
	Control		65	57 (95.0)	n/a	57	n/a	4	7.0	
mouse	SAHA 1000 nM/6 h	embryos	87	83 (98.0)	n/a	83	n/a	13	16.0	[125]
	Control		65	57 (95.0)	n/a	57	n/a	4	7.0	
mouse	Oxamflatin 1000 nM/6 h	embryos	117	106 (96.0)	n/a	106	n/a	8	7.5	[125]
	Control		233	229 (98.0)	n/a	229	n/a	6	2.6	
mouse	VPA 2 × 10^6^ nM/6 h	embryos	43	37 (95.0)	n/a	37	n/a	3	8.0	[125]
	Control		65	57 (95.0)	n/a	57	n/a	4	7.0	
mouse	Psammaplin A 1 × 10^4^ nM/16 h	embryos	303	252 (83.2)	131 (43.2)	224	n/a	4	1.8	[127]
	Control		207	196 (84.5)	40 (20.0)	216	n/a	1	0.5	
mouse	VPA 2 × 10^6^ nM/16 h	embryos	200	167 (83.5)	65 (32.5)	232	n/a	2	0.9	[127]
	Control		207	196 (84.5)	40 (20.0)	216	n/a	1	0.5	
mouse	Chlamydocin analogues, Ky-9 1600 nM/8 h	embryos	196	179 (91.3)	n/a	139	n/a	10	7.2	[141]
	Control		487	416 (85.4)	n/a	213	n/a	6	2.8	
mouse	*Xist* knockdown + TSA 50 nM/8 h	embryos	85	69 (81.0)	n/a	69	n/a	14	20.3	[203]
	Control		107	87 (81.0)	n/a	87	n/a	1	1.1	
mouse	*Xist* knockdown + TSA 50 nM/8 h	embryos	n/a	n/a	n/a	150	n/a	4	3.0	[204]
	Control		n/a	n/a	n/a	48	n/a	1	2.1	
mouse	Xist knockout	embryos	457	383 (84.0)	n/a	270	n/a	35	14.4	[200]
	Control		203	186 (91.5)	n/a	126	n/a	2	1.6	
mouse	*Kdm4d* mRNA injection	embryos	76	92.7%	88.6%	119	n/a	9	7.6	[116]
	Control		91	94.8%	26.0%	104	n/a	0	0	
mouse	*Xist* knockout + *Kdm4d* mRNA injection	donor cells + embryos	n/a	n/a	n/a	85	n/a	20	23.5	[196]
	Control		n/a	n/a	n/a	55	n/a	1	1.8	
mouse	*Kdm4b* + *Kdm5b* mRNA injection	oocytes	n/a	n/a	95.0%	n/a	n/a	n/a	11.1	[155]
	Control		n/a	n/a	31.0%	n/a	n/a	n/a	1.8	
mouse	*Dnmt3a/3b* siRNA and *Kdm4b/5b* mRNA injection	donor cells + embryos	119	n/a	92.6%	63	n/a	11	17.5	[180]
	Control		121	n/a	39.5%	247	n/a	2	0.8	
mouse	*Kdm6b* mRNA injection	embryos	286	89.1%	70.8%	265	n/a	16	6.0	[171]
	Control		176	90.9%	25.5%	120	n/a	0	0	
mouse	*Sfmbt2* miRNA knockout	donor cells	102	88 (86.3)	n/a	75	n/a	5	6.7	[195]
	Control		167	152 (91.0)	n/a	101	n/a	3	3.0	
mouse	Monoallelic deletion of *Sfmbt2*, *Jade1*, *Gab1*, *Smoc1*	donor cells	135	121 (89.6)	28 (23.0)	49	n/a	7	14.3	[173]
	Control		162	141 (87.0)	32 (21.7)	404	n/a	0	0	
mouse	RAD51-stimulatory compound 1: 1 × 10^4^ nM/22 h	embryos	169	159 (94)	119 (75)	171	n/a	8	4.7	[237]
	Control		154	144 (94)	45 (31)	146	n/a	1	0.6	
mouse	TSA 50 nM/8 h + vitamin C 5.7 × 10^4^ nM/7 h	embryos	61	n/a	51 (83.6)	105	n/a	16	15.2	[150]
	Control		94	n/a	34 (36.2)	178	n/a	0	0	
mouse	Vitamin C 1 × 10^5^ nM/16 h	embryos	194	174 (89.7)	101 (52.1)	206	n/a	8	3.9	[148]
	PsA 1 × 10^4^ nM/16 h		201	166 (82.6)	83 (41.3)	261	n/a	8	3.1	
	Vitamin C + PsA /16 h		193	164 (85.0)	109 (56.5)	203	n/a	10	4.9	
	Control		224	181 (80.8)	102 (45.5)	258	n/a	0	0	
mouse	*Dux* overexpression + DNMT3A/3B knockdown	embryos	56	51 (91.1)	n/a	43	n/a	8	18.6	[241]
	*Kdm4d* mRNA injection + DNMT3A/3B knockdown		58	54 (93.1)	n/a	49	n/a	6	12.2	
	Control		116	109 (94.0)	n/a	99	n/a	1	1.0	
cattle	TSA 50nM/10 h	embryos	237	222 (93.7)	103 (43.5)	36	6/13 (46.2)	3	8.3	[114]
	Control		198	193 (97.5)	63 (31.8)	18	0/7 (0)	0	0	
buffalo	DKK1 100 ng/mL	embryos	431	n/a	182 (42.6)	26	4/13 (30.8)	2	5.5	[234]
	Control		388	n/a	152 (39.0)	24	0/12 (0)	0	0	
cynomolgus monkey	*KDM4D* mRNA injection + TSA 10 nM/10 h	embryos	38	n/a	17 (44.7)	79	6/21 (28.6)	2	2.5	[103]
	Control		30	n/a	4 (13.8)	n/a	n/a	n/a	n/a	
minipig	Scriptaid 500 nM/14–16 h	embryos	155	134 (86.0)	33 (21.0)	1610	8/10 (80.0)	21	1.3	[118]
	Control		171	148 (87.0)	16 (9.0)	1389	1/9 (11.1)	3 mummies	0	
pig	Abexinostat 0.5 nM/6 h	embryos	106	94 (89.0)	27 (25.2)	782	1/3 (33.3)	2 fetuses	0	[128]
	Control		106	90 (85.0)	11 (10.2)	n/a	n/a	n/a	n/a	
pig	*XIST* knockout	donor cells	332	n/a	121 (36.4)	530	5/5 (100)	11	2.1	[164]
	Control		398	n/a	101 (25.4)	953	5/5 (100)	3	0.3	
pig	BIX-01294 50 nM/14–16 h	embryos	336	295 (87.8)	78 (23.2)	506	2/3 (66.7)	15	3.0	[167]
	Control		470	416 (88.5)	77 (16.4)	503	1/3 (33.3)	8	1.6	
pig	Vitamin C 2.8 × 10^5^ nM/15 h	embryos	74	71 (95.9)	25 (33.8)	5180	9/20 (45.0)	9	0.2	[146]
	Control		95	85 (89.5)	12 (12.6)	3885	5/15 (33.3)	1	0.03	
goat	*PGC7* overexpression	donor cells	n/a	n/a	n/a	300	14/30 (46.7)	21	7.0	[194]
	Control		n/a	n/a	n/a	240	8/24 (33.3)	8	3.3	
